# Neural Tuning for Ordinal Processing: Convergent Patterns in Human Brains and Artificial Networks

**DOI:** 10.1523/JNEUROSCI.1237-25.2026

**Published:** 2026-02-11

**Authors:** Shir Hofstetter, Marcus Daghlian, Serge O. Dumoulin

**Affiliations:** ^1^Spinoza Centre for Neuroimaging, Amsterdam 1105 BK, Netherlands; ^2^Computational Cognitive Neuroscience and Neuroimaging, Netherlands Institute for Neuroscience, Amsterdam 1105 BA, Netherlands; ^3^Experimental and Applied Psychology, Vrije University Amsterdam, Amsterdam 1081 HV, Netherlands; ^4^Experimental Psychology, Utrecht University, Utrecht 3584 CS, Netherlands

**Keywords:** convolutional neural network, fMRI, neural tuning, ordinality, perception, population receptive fields

## Abstract

Processing ordinality, i.e., the rank of an item in a series such as 1st, 2nd, 3rd, etc., is a fundamental skill shared by humans and animals. While humans often use symbolic sequences like numbers or letters, ordinality does not depend on language or symbols. Across species, ordinality plays a critical role in behaviors such as decision-making, foraging, and social organization. We hypothesize that ordinality perception is supported by neuronal tuning, i.e., neurons selectively responsive to specific ranks. Using ultrahigh-field 7 T fMRI and population receptive field (pRF) modeling in human participants (both female and male), we identified neural populations in parietal and premotor cortices that are tuned to nonsymbolic ordinal positions. Comparable with other sensory domains, tuning width increased with preferred ordinal rank, suggesting reduced precision and potentially lower perceptual accuracy for higher ranks. Additionally, pRF measurements revealed that cortical territory devoted to higher ordinalities decreased with rank, reinforcing that neural precision is greatest for early positions (e.g., 1st and 2nd) and declines with rank. These responses did not generalize to symbolic ordinality. Similar tuning to nonsymbolic ordinality emerged spontaneously in hierarchical convolutional neural networks trained on visual tasks. Together, these results suggest that the tuning properties of these neuronal populations support nonsymbolic ordinality perception and may reflect an inherent feature of neural processing.

## Significance Statement

Processing ordinality, the rank of items in sequences, is a fundamental skill shared across humans and animals that plays a role in decision-making, foraging, and social organization. We hypothesized that ordinality processing relies on neuronal tuning where neurons selectively respond to particular ranks. Using ultrahigh-field 7 T fMRI and population receptive field modeling, we identified neural populations in parietal and premotor cortices tuned to nonsymbolic ordinal positions. Additionally, similar tuning responses were found to spontaneously emerge in hierarchical convolutional neural networks trained on a visual task. Our findings demonstrate that akin to other forms of quantity representation, neuronal tuning underlies nonsymbolic ordinality perception. These results shed light on the neuronal processing of ordinality in the human brain.

## Introduction

Identifying ordinal relationships, e.g., recognizing the 1st, 2nd, or 5th item in a sequence, is a widespread and essential cognitive ability shared by humans and animals. While humans often rely on symbolic systems like numbers, letters, or days of the week to represent ordinality, ordinality processing does not depend on symbolic or linguistic representations (Brannon, 2002; Suanda et al., 2008; Macchi Cassia et al., 2012). Across the animal kingdom, from fish (Petrazzini et al., 2015; Potrich et al., 2019) to bees, birds (Rugani et al., 2008; Pfuhl and Biegler, 2012; Vámos et al., 2020), rats (Davis and Bradford, 2010), chicks (Rugani et al., 2007), and monkeys (Chen et al., 1997; Brannon and Terrace, 1998; Orlov et al., 2000; Brannon et al., 2006), ordinal information guides behavior, including decision-making, foraging, and social hierarchies.

How does the brain encode ordinality? We hypothesize that ordinality perception relies on neuronal tuning, where neurons respond selectively to a specific range of a stimulus domain. Tuning is a fundamental neuronal property underlying perception. For example, neurons in visual cortex are tuned to specific locations in the visual field (Inouye, 1909; Holmes, 1918). Neuronal tuning is not limited to locations on sensory organs, e.g., visual neurons are also tuned to specific orientations, spatial frequencies, and motion directions. Perception of these features depends on the activity of correspondingly tuned neurons (Hubel and Wiesel, 1977; Salzman et al., 1990; Tsouli et al., 2022). Moreover, tuned neuronal responses are also found for several types of magnitude perception such as numerosity (Nieder et al., 2002; Piazza et al., 2007; Harvey et al., 2013; Ditz and Nieder, 2016; Kobylkov et al., 2022), object size (Harvey et al., 2015), and visual timing (Protopapa et al., 2019; Harvey et al., 2020). Notably, tuning characteristics are suggested to underlie the perception of these quantities (Nieder, 2016; Tsouli et al., 2022).

Here, we ask whether similar tuning mechanisms support ordinality perception: are there neuronal populations selectively responsive to specific ranks in a sequence, and whether they respond similarly to nonsymbolic and symbolic ordinal stimuli? To address this, we combined ultrahigh-field 7 T fMRI with population receptive field (pRF) modeling to examine neural responses to ordinal position (Dumoulin and Wandell, 2008; Harvey et al., 2013; Cai et al., 2023). Complementing our neuroimaging data, we also investigated whether ordinality-tuned units emerge in a biologically inspired hierarchical convolutional neural network (HCNN). HCNNs have achieved notable success in modeling the brain's vision (Cadieu et al., 2014; Dobs et al., 2022) and numerosity processing (Nasr et al., 2019; Kim et al., 2021). Mirroring the brain's architecture, HCNNs are composed of feedforward and retinotopically organized layers that emulate different types of visual neurons (Lecun and Bengio, 1995; Kriegeskorte, 2015). This dual human neuroimaging and neural network approach allows us to explore both biological and artificial systems for converging evidence of a representational basis for ordinality.

## Materials and Methods

### Study 1: fMRI experiment

#### Participants

Ten participants took part in the study (four females, two left-handed; mean age, 32; age range, 23–48). All participants had normal or corrected-to-normal visual acuity. All experimental procedures were approved by the Scientific and Ethical Review Board of the Faculty of Behavioral and Movement Sciences of the Vrije University Amsterdam (VCWE-2020-004). Participants gave informed consent.

**Figure 1.**
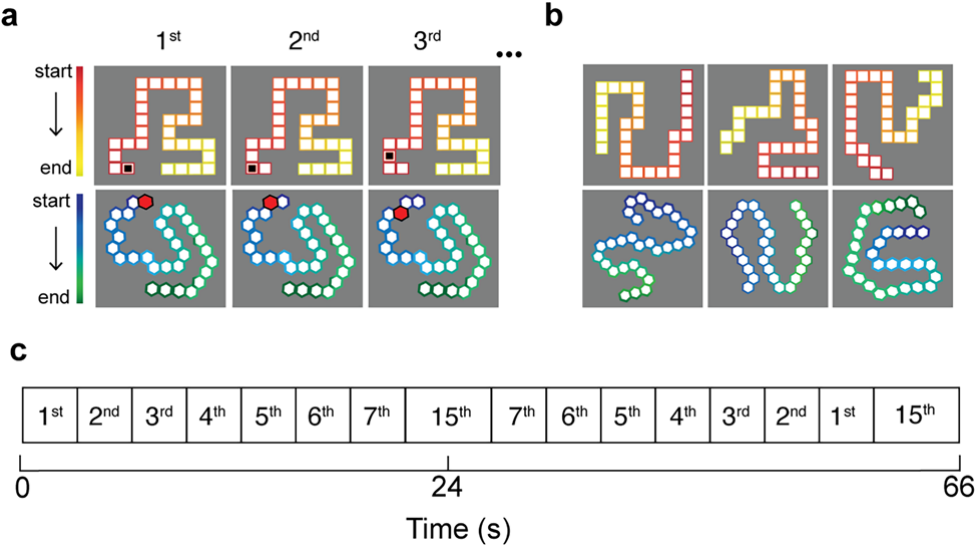
fMRI nonsymbolic experimental stimuli. Images show the two stimulus conditions: square or polygons “snakes”. ***a***, A flickering black or red marker moved along the rectangular or polygons snakes from the first to the seventh position. The middle point of the snake served as baseline, i.e., the 15th position. The directionality of the snake was indicated by a colour gradient. ***b***, At each fMRI run the snake was presented in a different spatial layout and a few example layouts are shown in panel ***b***. Every participant viewed eight different layouts of the two different types of snakes. Thus, to show that the results do not depend on the spatial configuration, e.g., shape or color, we used two types of snakes each with eight different layouts resulting in 16 different spatial layouts. ***c***, The marker moved along the snake in consecutive steps. This cycle was repeated four times per functional run.

*Stimuli and experiment design*. *Nonsymbolic ordinality*. The participants viewed images of elongated “snakes” comprising 30 rectangles or polygons. The directionality of the snake, i.e., start and end points, was represented using a colored gradient, e.g., from red to yellow in the snake comprised of rectangles (Fig. 1). Images were presented in a 2° radius to minimize eye movements. To show that the results do not depend on spatial configuration, e.g., spatial locations or relative location, or other stimulus confounds, e.g., shape or color, the two types of stimuli shapes were presented with eight different spatial layouts (Fig. 1*b*). At each functional run, the snake was presented in a different configuration in visual space. Participants viewed a total of 16 configurations (square and polygons) in two scanning sessions

To point to specific ordinalities along the snake, a marker moved systematically, i.e., both in ascending and descending order along the first to seventh positions. In one cycle, the marker moved in an ascending order, followed by 12 s showing the marker in the middle position (15th position), followed by a descending order from the 7th position to the first position, followed by a similar presentation of the 15th position for 12 s (Fig. 1*c*). This cycle was repeated four times during a functional run. In each position the marker was presented for 30 ms followed by 20 ms of gray. In each of the 1st to 7th positions, the marker was shown six times over 2 TRs (3 s) before moving to the next position. Once the marker completed pointing to the first seven positions, it was placed in the middle position of the snake (15th position; Fig. 1*c*). This position served as baseline in the pRF analysis.

The task did not ask for direct attention to the marker specific position or ordinality, but the participants were asked to respond when the marker moved one step forward along the snake (Cai et al., 2023; i.e., from 2nd to 3rd). This happened at regular intervals during the ascending sequence but also randomly, on four time points in each cycle, during the descending sequence. In other words, the marker could move a step forward even in the descending sequence where the marker returned from the 7th position the 1st. No ordinality judgment was required. This task is akin to numerosity mapping, where often a color judgment is required but without explicit numerosity judgment (Harvey et al., 2013; Harvey and Dumoulin, 2017). Attention to the stimulus alone is enough for the brain to encode numerosity (Cai et al., 2022). Furthermore, this task avoids the confound that difficulty of numerosity judgments vary with numerosity (which also holds for ordinality). Thus, we assume attention to the stimulus is enough for the brain to encode ordinality, and no ordinality judgment are needed.

Stimuli were presented on a 69.84 × 39.29 cm LCD screen (Cambridge Research Systems) at the end of the MRI bore and viewed through a mirror. The total distance from the subject's eyes was 220 cm. The display resolution was 1,920 × 1,080 pixels. Stimuli images were generated in illustrator and displayed using MATLAB and Psychtoolbox (Brainard, 1997; Pelli, 1997).

*Symbolic ordinality: spatial alphabet.* The alphabet letters were presented in black on a gray background in their learned order from left to right across several rows **(**Fig. S3*a***)**. The letters were either all small or all capital letters. The spatial position of the letters on the background was different in each functional run. Overall, eight types of ABC letters configurations were made, and their display order was randomized. Letters from A to G were marked in red in an ascending order, followed by a red presentation of the letter M, followed by a descending order of the letters G to A, followed by a similar presentation of the letter M in red. The cycle timing and number of repetitions per letter was similar to the marker movement along the snake shapes.

Participants were asked to press a button when the following letter of the ABC was marked. As the marked letters were presented in an ascending order, there were always seven regular stimulus changes in one cycle that would require a response. At random points in the cycle, we added four additional events where the next letter was marked. No ordinality judgment was required (Text S1).

*Symbolic ordinality: temporal alphabet.* Six out of the 10 participants took part in this experiment (four females, one left-handed; mean age, 30; age range, 23–48). Here, the alphabet letters were presented sequentially in black on a gray background in their learned order. The letters were either all small or all capital letters. Letters from A to G were presented in an ascending order, followed by a presentation of the letter M, followed by a descending order of the letters G to A, followed by a similar presentation of the letter M. The cycle timing and number of repetitions per letter was similar to the previous conditions. The stimuli were generated and displayed using Psychtoolbox (Brainard, 1997; Pelli, 1997).

Participants were asked to press a button when the following letter of the ABC was shown. As the letters were presented in an ascending order, there were always seven regular stimulus changes in one cycle that would require a response. At random points in the cycle, we added four additional events where the next letter was marked. No ordinality judgment was required (Text S2).

*MRI acquisition and preprocessing.* MRI data was acquired on a 7 T Philips Achieva scanner. T1-weighted images were acquired using MP2RAGE sequence with the following parameters: TR, 6.2 ms; TE, 2.5 ms; flip angle, 8°; isotropic resolution of 0.7^3^; SENSE (AP), 1.8; (RL), 1.8; slices, 265.

Functional runs were acquired using a 32 channel head coil with the following parameters: isotropic resolution of 1.7 mm^3^; TR/TE, 1,500/22 functional scans, respectively; flip angle, 65; multiband factor, 3, 57 slices. The functional runs included 184 TRs and lasted 276 s.

The experiments included eight repeated runs in each scanning session. Each stimulus configuration (rectangular, polygon or ABC letters) was acquired in one scanning session on different days.

Denoising of scans was applied to the functional runs using NORDIC PCA (Moeller et al., 2021; Vizioli et al., 2021). Next, the preprocessing was performed using *fMRIPrep* 20.2.3 (Esteban et al., 2019) which is based on *Nipype* 1.6.1 (Gorgolewski et al., 2011, 2017).

#### Anatomical data preprocessing

The T1-weighted (T1w) image was corrected for intensity nonuniformity (INU) with N4BiasFieldCorrection (Tustison et al., 2010), distributed with ANTs 2.3.3 (Avants et al., 2008), and used as T1w reference throughout the workflow. The T1w reference was then skull-stripped with a *Nipype* implementation of the antsBrainExtraction.sh workflow (from ANTs), using OASIS30ANTs as target template. Brain tissue segmentation of cerebrospinal fluid (CSF), white matter (WM), and gray matter (GM) was performed on the brain-extracted T1w using fast (FSL 5.0.9; Zhang et al., 2001). Brain surfaces were reconstructed using recon-all (FreeSurfer 6.0.1; Dale et al., 1999), and the brain mask estimated previously was refined with a custom variation of the method to reconcile ANTs-derived and FreeSurfer-derived segmentations of the cortical gray matter of Mindboggle (Klein et al., 2017). Volume-based spatial normalization to one standard space (MNI152NLin2009cAsym) was performed through nonlinear registration with antsRegistration (ANTs 2.3.3), using brain-extracted versions of both T1w reference and the T1w template. The following template was selected for spatial normalization: *ICBM 152 Nonlinear Asymmetrical template version 2009c* (Fonov et al., 2009; TemplateFlow ID: MNI152NLin2009cAsym).

#### Functional data preprocessing

For each of the BOLD runs found per subject (across all tasks and sessions), the following preprocessing was performed. First, a reference volume and its skull-stripped version were generated using a custom methodology of *fMRIPrep*. A deformation field to correct for susceptibility distortions was estimated based on *fMRIPrep*'s *fieldmap-less* approach. The deformation field is that resulting from coregistering the BOLD reference to the same-subject T1w reference with its intensity inverted (Huntenburg, 2014; Wang et al., 2017). Registration is performed with antsRegistration (ANTs 2.3.3), and the process regularized by constraining deformation to be nonzero only along the phase-encoding direction, and modulated with an average fieldmap template (Treiber et al., 2016). Based on the estimated susceptibility distortion, a corrected EPI (echo-planar imaging) reference was calculated for a more accurate coregistration with the anatomical reference. The BOLD reference was then coregistered to the T1w reference using bbregister (FreeSurfer) which implements boundary-based registration (Greve and Fischl, 2009). Coregistration was configured with six degrees of freedom. Head-motion parameters with respect to the BOLD reference (transformation matrices and six corresponding rotation and translation parameters) are estimated before any spatiotemporal filtering using mcflirt (FSL 5.0.9; Jenkinson et al., 2002). The BOLD time series were resampled onto the following surfaces (FreeSurfer reconstruction nomenclature): *fsnative*, *fsaverage*. The BOLD time series (including slice-timing correction when applied) were resampled onto their original, native space by applying a single, composite transform to correct for head-motion and susceptibility distortions. These resampled BOLD time series will be referred to as *preprocessed BOLD in original space* or just *preprocessed BOLD*. The BOLD time series were resampled into standard space, generating a *preprocessed BOLD run in MNI152NLin2009cAsym space*. First, a reference volume and its skull-stripped version were generated using a custom methodology of *fMRIPrep*. Several confounding time series were calculated based on the *preprocessed BOLD*: framewise displacement (FD), DVARS, and three region-wise global signals. FD was computed using two formulations following Power (absolute sum of relative motions; Power et al., 2014) and Jenkinson (relative root mean square displacement between affines; Jenkinson et al., 2002). FD and DVARS are calculated for each functional run, both using their implementations in *Nipype* (following the definitions by Power et al., 2014). The three global signals are extracted within the CSF, the WM, and the whole-brain masks. Additionally, a set of physiological regressors were extracted to allow for component-based noise correction (*CompCor*; Behzadi et al., 2007). Principal components are estimated after high-pass filtering the *preprocessed BOLD* time series (using a discrete cosine filter with 128 s cutoff) for the two *CompCor* variants: temporal (tCompCor) and anatomical (aCompCor). tCompCor components are then calculated from the top 2% variable voxels within the brain mask. For aCompCor, three probabilistic masks (CSF, WM and combined CSF + WM) are generated in anatomical space. The implementation differs from that of Behzadi et al. in that instead of eroding the masks by 2 pixels on BOLD space, the aCompCor masks are subtracted a mask of pixels that likely contain a volume fraction of GM. This mask is obtained by dilating a GM mask extracted from the FreeSurfer's *aseg* segmentation, and it ensures components are not extracted from voxels containing a minimal fraction of GM. Finally, these masks are resampled into BOLD space and binarized by thresholding at 0.99 (as in the original implementation). Components are also calculated separately within the WM and CSF masks. For each CompCor decomposition, the *k* components with the largest singular values are retained, such that the retained components' time series are sufficient to explain 50% of variance across the nuisance mask (CSF, WM, combined, or temporal). The remaining components are dropped from consideration. The head-motion estimates calculated in the correction step were also placed within the corresponding confounds file. The confound time series derived from head motion estimates and global signals were expanded with the inclusion of temporal derivatives and quadratic terms for each (Satterthwaite et al., 2013). Frames that exceeded a threshold of 0.5 mm FD or 1.5 standardized DVARS were annotated as motion outliers. All resamplings can be performed with a single interpolation step by composing all the pertinent transformations (i.e., head-motion transform matrices, susceptibility distortion correction when available, and coregistrations to anatomical and output spaces). Gridded (volumetric) resamplings were performed using antsApplyTransforms (ANTs), configured with Lanczos interpolation to minimize the smoothing effects of other kernels (Lanczos, 1964). Nongridded (surface) resamplings were performed using mri_vol2surf (FreeSurfer).

#### fMRI data analysis

pRF modeling was applied to estimate ordinality responses, similar to what was previously described for numerosity studies (Dumoulin and Wandell, 2008; Harvey et al., 2013; Harvey and Dumoulin, 2017; Hofstetter et al., 2021). Briefly, the pRF model describes the averaged tuning of the underlying neuronal populations using logarithmic Gaussian functions that are characterized by preferred ordinality (mean of the Gaussian) and tuning width (standard deviation of the Gaussian).

At each voxel in the cortical gray matter, the pRF model is estimated based on the fMRI data and the time course of presented ordinalities. For each candidate preferred ordinality and tuning width, a predicted neuronal response time course is calculated as the amplitude of the candidate neuronal response function at each time point's presented ordinality. Each candidate predicted neuronal response time course is then convolved with the hemodynamic response function (HRF) to create a candidate predicted fMRI time course. The chosen pRF parameters for each voxel are those whose predicted fMRI time course is best correlated with the voxel's measured fMRI time course. Last, subject-specific HRF parameters were estimated over the whole fMRI volume (Harvey and Dumoulin, 2011). These parameters were used to refit the pRF.

The pRF fitting procedure allows preferred ordinality estimates outside the range of the presented stimuli, ensuring estimates within the stimulus range are not just the best of a limited set. We excluded from analysis any recording sites where the preferred ordinality was outside our presented range.

The pRF model was calculated in VistaSoft (https://github.com/vistalab/vistasoft/wiki) using the anatomical images and the registered functional runs. First, the first eight timeframes were discarded from the functional scans. The time series data were aligned to the T1-weighted anatomical space and then averaged based on each functional condition.

Segmented anatomical maps were created using the cortical ribbon images created in FreeSurfer. Segmentation errors were manually edited using ITK-SNAP (Yushkevich et al., 2006). The cortical surface was reconstructed at the gray-white matter border and rendered as a smoothed 3D surface.

Functional data were interpolated to the anatomical segmentation space using a trilinear interpolation. To increase signal strength, data from all recording points (voxels) were collapsed and averaged onto the nearest point on the cortical surface. This formed a (folded) two-dimensional representation of the gray matter nodes and increased signal strength (Harvey et al., 2013). The statistics were adjusted to account for upsampling from the acquired data to the anatomical space. No spatial or temporal smoothing was applied to the functional data.

#### Cross-validation

We estimated the goodness of fit of the pRF prediction from one nonsymbolic condition (rectangular or polygon snakes) to the time course of the other, yielding new values of goodness of fit. Voxels where the averaged variance explained of the cross-validated datasets was lower than 20% were excluded from further analysis.

We also fitted the pRF model estimated on both nonsymbolic conditions to the time series of the symbolic conditions. Voxels where the variance explained by the nonsymbolic pRF model was lower than 20% were excluded from further analysis.

#### Analysis within cortical regions

We calculated the significance of the tuning width change with preferred ordinality by combining the data points in each cortical area (parietal or premotor) across participants. We ran Spearman rank test, followed by permutation test (10,000 permutations) to account for upsampling of the original data points.

We assessed the cortical surface area preferring specific ordinality range. In each cortical area, for each participant, we binned the recording points from 1 to 7 and divided each bin by the overall size of the measured area.

#### Combined presentation of maps in MNI space

In order to show the spatial overlap between the model fits across subjects, we also analyzed each participant's data in MNI space using the functional and anatomical scans that were normalized to MNI space in the fmriprep pipeline. We fitted the pRF model and ran a cross-validation analysis for each participant as described before. Finally, we combined into one map all of the participants' individual pRF maps of the averaged shape conditions (rectangular and polygon snakes) that showed preferred ordinality between 1 and 7 with a variance explained that exceeded 20% in the cross-validation analysis.

### Study 2: neural network model

#### CNN model architecture and training

We implemented an HCNN, a standard CNN architecture that was previously shown to be successful in image classification (Russakovsky et al., 2015; Nasr et al., 2019). Our model consisted of eight convolutional layers, each followed by batch normalization and max-pooling (Table 1). The model was trained for 25 epochs using stochastic gradient descent (SGD) with a learning rate of 0.1, a batch size of 256, and ReLU activation function, with cross-entropy loss function as the training objective.

**Table 1.** The structure of the HCNN model: the HCNN model consists of eight convolutional blocks

#	Type	# Feature maps	Spatial size	Kernel size
–	Input	3	224 × 224	–
1	Conv2d	32	224 × 224	9 × 9
2	BatchNorm2d	32	224 × 224	–
3	MaxPool2d	32	112 × 112	–
4	Conv2d	48	112 × 112	9 × 9
5	BatchNorm2d	48	112 × 112	–
6	MaxPool2d	48	56 × 56	–
7	Conv2d	96	56 × 56	7 × 7
8	BatchNorm2d	96	56 × 56	–
9	MaxPool2d	96	28 × 28	–
10	Conv2d	192	28 × 28	5 × 5
11	BatchNorm2d	192	28 × 28	–
12	MaxPool2d	192	14 × 14	–
13	Conv2d	384	14 × 14	5 × 5
14	BatchNorm2d	384	14 × 14	–
15	MaxPool2d	384	7 × 7	–
16	Conv2d	768	7 × 7	5 × 5
17	BatchNorm2d	768	7 × 7	–
18	Conv2d	768	7 × 7	5 × 5
19	BatchNorm2d	768	7 × 7	–
20	Conv2d	768	7 × 7	5 × 5
21	BatchNorm2d	768	7 × 7	–
22	AdaptiveAvgPool2d	768	1 × 1	–
23	Linear	1,000	1 × 1,000	–

Each block contains a convolutional layer followed by batch normalization and a ReLU activation function. Max-pooling is applied after the first five of these blocks. Following the convolutional layers, an adaptive average pooling layer is applied, and a fully connected layer then computes the scores for each class.

The input to the model was colored images of size 224 × 224 pixels from the ILSVRC2012 ImageNet dataset which encompasses images from 1,000 distinct object categories (Russakovsky et al., 2015). The training dataset comprised ∼1.2 million images, where each image was labeled with its primary object category. The model's object classification accuracy was assessed on a separate set of 50,000 unseen images (Russakovsky et al., 2015).

**Figure 2.**
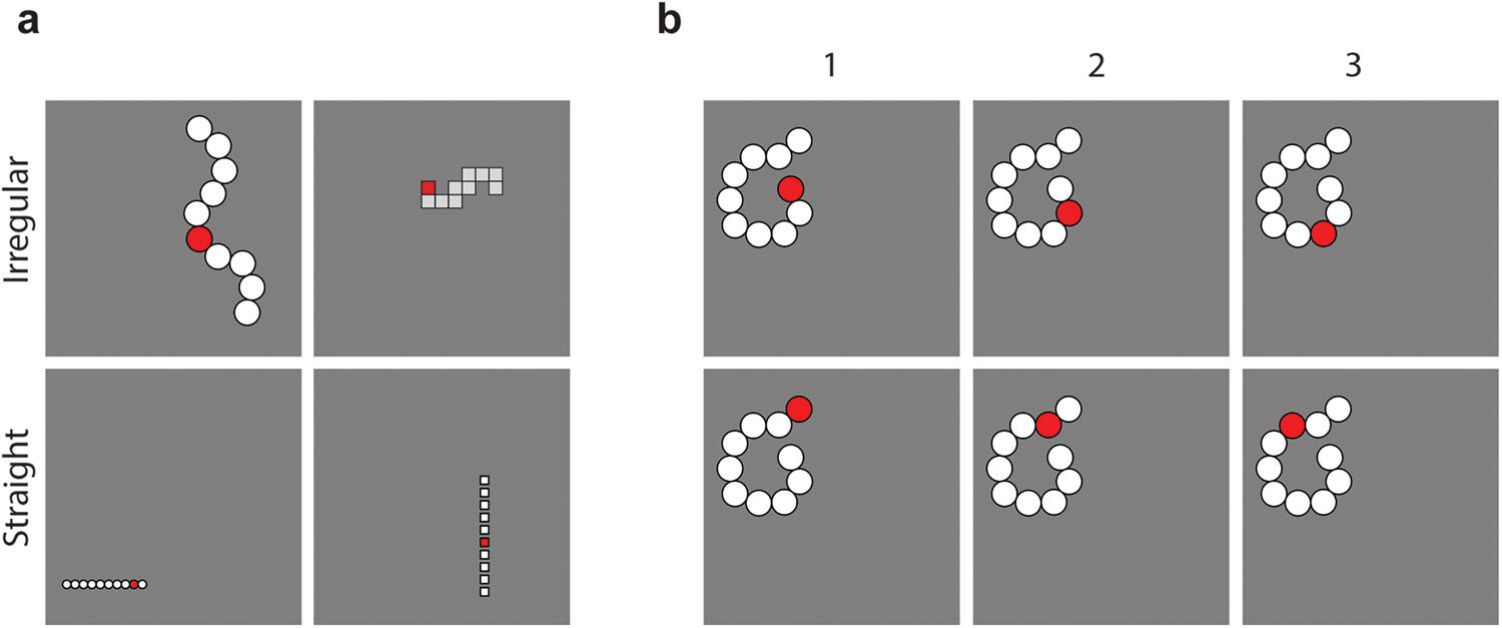
Stimuli for HCNN experiment. ***a***, Images show the two stimulus conditions: irregular or straight “snakes.” A red marker indicated an ordinality within the snake. ***b***, Given no directionality were provided, the red maker could represent the same ordinality from both ends of the snakes.

*Ordinality stimuli*. After training on object classification, the HCNN was presented with images of “snake” stimuli, which were computer-generated constructs of 10 white rectangles or circles on a gray background (Fig. 2*a*). A red marker was placed within one of the snake's segments. Given the absence of directional cues (i.e., no “beginning” or “end” to the snake shape), the same ordinality was determined from both ends of the snakes, yielding a maximum ordinality of 5. (Fig. 2*b*). The snakes varied in their spatial position and were created randomly. The stimulus dataset consisted of two categories:

1. Straight line snakes: These were generated through 5 repetitions of all combinations of 2 shapes (circles/rectangles), 2 orientations (horizontal/vertical), 2 interval types (spaced/uninterrupted), and 10 marker positions (corresponding to 5 ordinalities from both ends), resulting in 400 images (80 per ordinality).
2. Irregularly ordered snakes: These were produced with 20 repetitions of combinations of 2 shapes (circles/rectangles) and 10 marker positions (representing 5 ordinalities), yielding 400 images (80 per ordinality).

We further evaluated the tuned responses of the neural units using another set of images featuring longer shapes with either 16 or 20 compartments. The 16-compartment shapes (representing 8 ordinalities) consisted of 640 images with irregular order (20 repetitions × 16 positions × 2 shapes) and 640 images with straight order (5 repetitions × 2 shapes × 2 orientations × 2 spacing conditions × 16 positions). The 20-compartment shapes (representing 10 ordinalities) included 800 images with irregular order (20 repetitions × 20 positions × 2 shapes) and 800 images with straight order (5 repetitions × 2 shapes × 2 orientations × 2 spacing conditions × 20 positions).

Lastly, we tested the neural unit responses using two additional sets of stimuli. The first set consisted of irregularly ordered snakes with varied compartment lengths, designed to control for the potential confound between ordinality and the distance from the stimulus's starting point (Text S3, Fig. S6*a*; *N* = 200: 20 repetitions × 10 positions corresponding to 5 ordinalities from both ends). We then assessed the reliability of the tuning patterns by presenting the neural units with the fMRI stimuli (Text S3), allowing for a direct comparison with the human neuroimaging data.

All ordinality stimuli were preprocessed in the same way as the ImageNet stimuli to enable inference of the network's responses. Preprocessing included resizing and cropping the images to 224 × 224 pixels, followed by normalization.

*Statistical analysis*. We analyzed the final layer's responses to the presented images using a two-way ANOVA with ordinality and stimulus set as factors. This analysis was performed on 37,632 units using a Python script.

## Results

### Ordinality-tuned neuronal populations in the cortex

**Figure 3.**
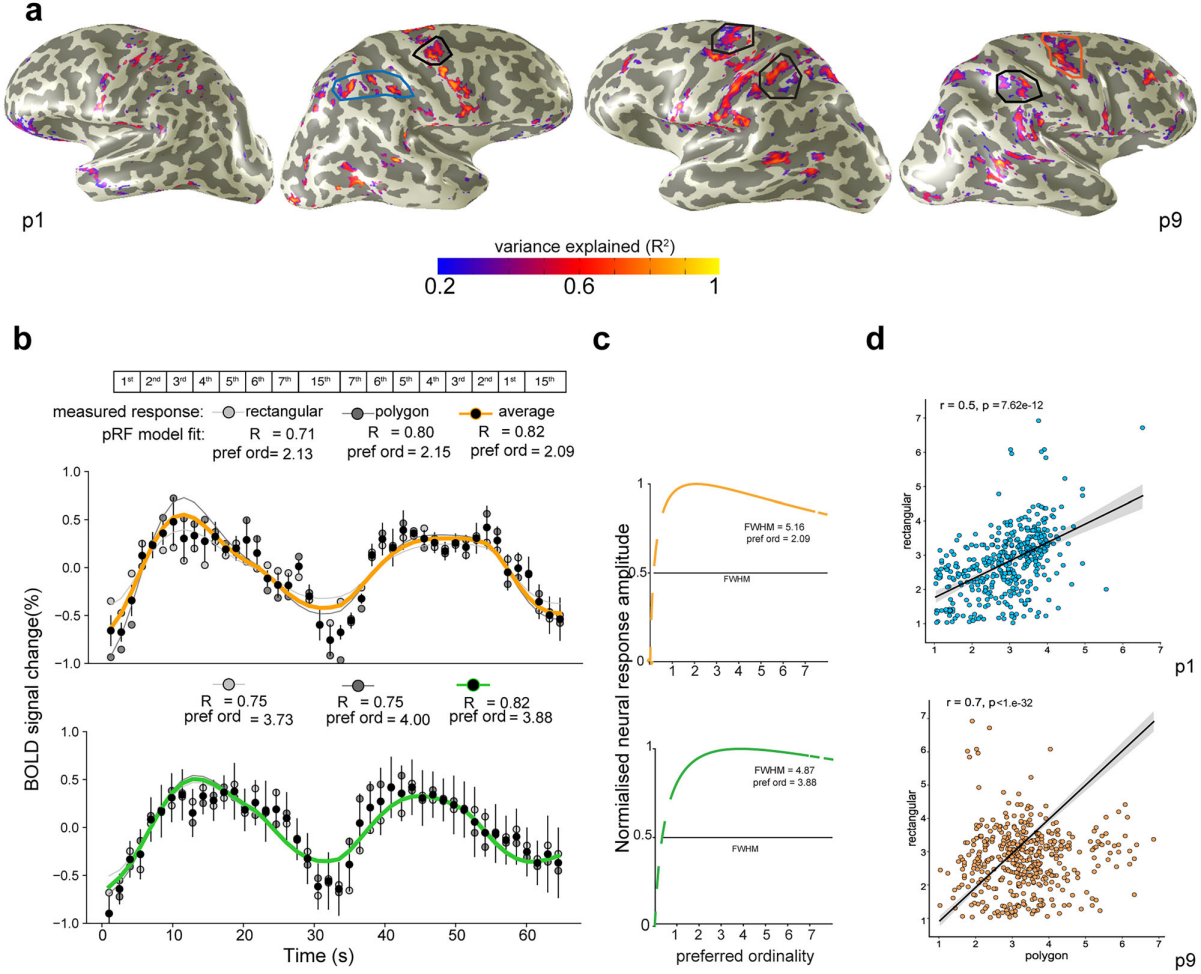
Ordinality-tuned responses to nonsymbolic ordinality. ***a***, Variance explained (goodness of fit) of ordinality-tuned pRF models are shown on the cortical surfaces of two example participants (p1 and p9). The cross-validated variance (*R*^2^) explained by the ordinality model is derived across the two stimulus conditions (Fig. 1*a*,*b*). The lines define the borders of clusters along the intraparietal sulcus and the premotor cortex. ***b***, Two example of fMRI time courses are shown from the cortical locations to the right intraparietal sulcus of participant 1 (blue box in ***a***). The two cortical locations show different responses to different ordinalities. The ordinality pRF model's predictions (solid lines) capture the different responses at different ordinality selectivity parameters. The two types of stimuli (rectangular or polygon “snakes”) elicit similar fMRI responses. ***c***, The pRF model summarizes the neuronal responses using a logarithmic Gaussian tuning function with two parameters: preferred ordinality and tuning width, defined by the full-width at half-maximum (FWHM). The two cortical locations prefer different ordinalities. ***d***, Preferred ordinality in clusters along the intraparietal sulcus and the premotor area were well correlated between stimulus configurations, demonstrating repeatable tuned responses across stimulus conditions.

We found that the pRF model explained a large amount of the signal variance particularly along the intraparietal sulcus and the premotor cortex. Eight of our 10 participants showed significant cross-validated responses in these cortices (averaged variance explained in the parietal and premotor clusters = 53%; Fig. 3*a* and for all participants see Fig. S1*a*). Since these were the areas that showed significant cross-validated responses in most participants, our next analyses focus on the tuned responses in these areas.

Within the parietal and premotor regions, the pRF model reveals neuronal populations tuned to ordinality (Fig. 3*b*,*c*, for example, individual cortical locations; for mean tuning curves per ordinality see Suppl. Fig. S2). As expected based on the cross-validation analyses, in the clusters along the intraparietal sulcus and the premotor cortex preferred ordinality was well correlated between stimulus configurations (Fig. 3*d*). The correlation between stimulus conditions was significant in 10 of the 12 (83.3%) defined clusters along the intraparietal sulcus and in all of the clusters defined in the premotor area (FDR corrected for multiple comparisons), demonstrating repeatable tuned responses across stimulus conditions.

**Figure 4.**
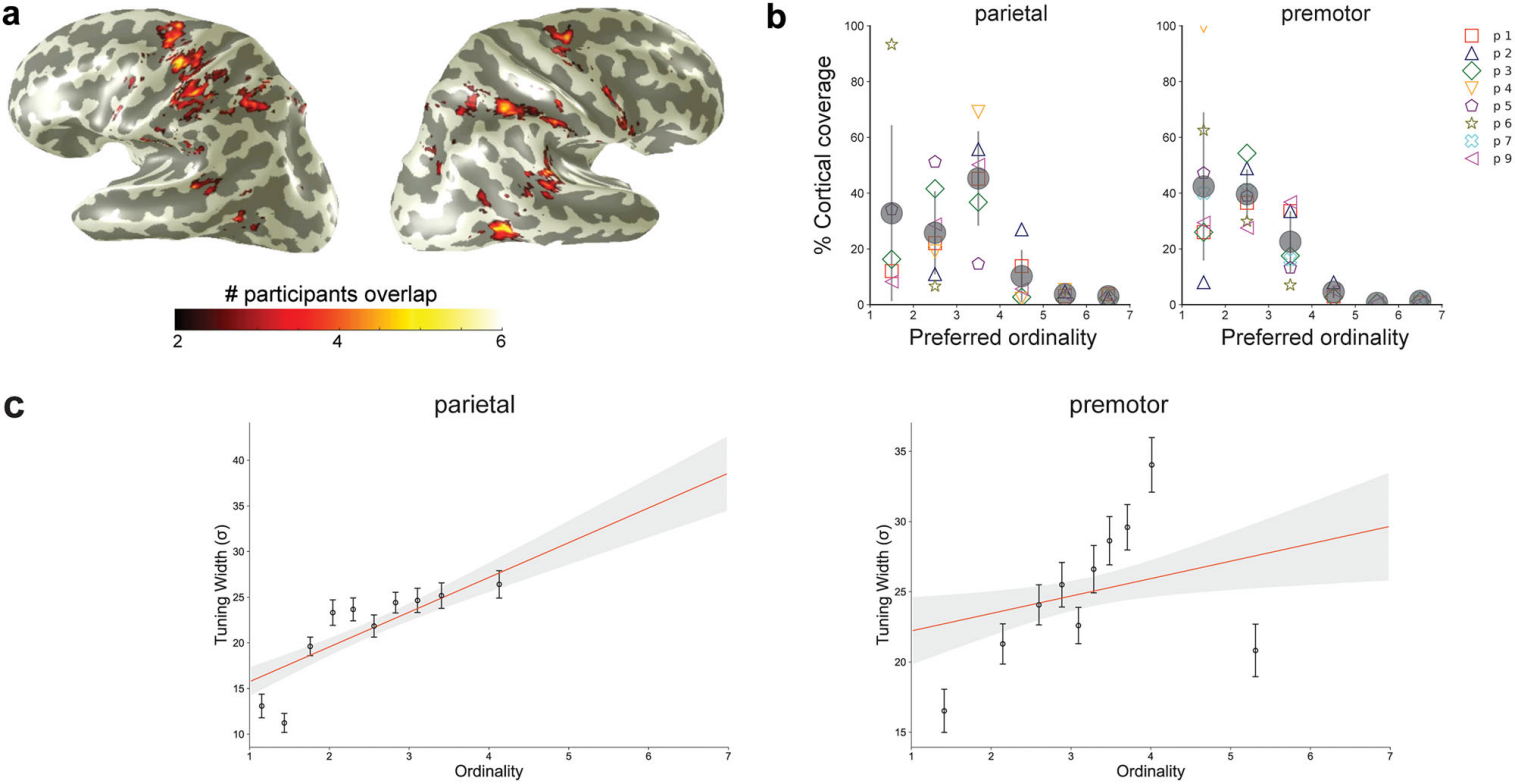
***a***, Ordinality responses of all participants transformed onto the MNI template's cortical surface anatomy reveal consistent ordinality-tuned neuronal populations predominantly in intraparietal sulcus and premotor cortex. Colors indicate the overlap between the maps of the participants. ***b***, Percentage coverage of the clusters in the premotor and parietal cortices plotted as a function of preferred ordinality, grouped across hemispheres. The distribution of preferred ordinality reveals that most of the measured cortical points prefer the first four ordinalities. Black circles show the averaged cortical coverage across participants. Error bars show the standard deviation of the mean. ***c***, Increase in tuning widths as a function of the preferred ordinality, grouped across participants. For visual clarity, the data points were divided into 10 equal-sized bins, and the bars show the average and standard error for the points within each bin. Red line shows a linear regression fit with their 95% confidence intervals (gray).

Combining the participants further highlighted the intraparietal sulcus and the premotor cortex (Fig. 4*a*). The ordinality-tuned neuronal populations did not show a clear systematic topographic order (Fig. S1*b*), and the vast majority of cortical locations were found to represent the lower range of the marked positions (up to 4; Fig. 4*b*). Furthermore, at the group level, we found a significant increase in tuning width with preferred ordinality in each of the cortical areas (Spearman rank test and permutation analysis, one tail; parietal cortex: ρ = 0.2764 *p* = 0.0001, premotor cortex: ρ = 0.0565 *p* = 0.0022; Fig. 4*c*).

### Ordinality tuning does not generalize to symbolic ordinality

Next, we asked whether the ordinality-tuned neuronal populations generalize to symbolic representations of ordinality, specifically the alphabetical letters. We measured fMRI responses while the participants viewed either all the alphabet letters in one spatial representation (“spatial alphabet”) or where they were presented sequentially (“temporal alphabet”; Text S1–2; Figs. S3–S5). In the spatial alphabet configuration, the target letter changed color. In both cases, the target letters were representing the same ordinality and were presented in a similar sequence as the nonsymbolic ordinality (Fig. 1*c*; Fig. S3*a*,*b*). Although we find evidence for tuning in the spatial alphabet configuration, we do not find evidence in the temporal alphabet configuration (Fig. S5).

We speculate that ordinality tuning of the spatial alphabet may be driven by a nonsymbolic mechanism. Specifically, the simultaneous presentation could be interpreted as a directional snake-like visual path. In other words, since the letters are presented in a highly predictable manner, the spatial alphabet could be treated as a purely visual task minimizing the need for symbolic reading. However, a symbolic mechanism cannot be ruled out and different participants may have followed different strategies. Finally, the neuronal responses to the temporal alphabet condition were evaluated against the responses to the sequential nonsymbolic task. This evaluation means that a possible effect of the temporal feature of the task on the neural responses is also included in the comparison. However, even if the temporal aspect of the task influences the neural response, an abstract neural representation to stimulus type (symbolic and nonsymbolic) should have been revealed. Given that we only found evidence of tuning in the spatial but not the temporal alphabet condition, these results do not support the hypothesis that tuned responses to nonsymbolic ordinality generalize to symbolic ordinality.

### Ordinality tuning in artificial neural networks

We trained an HCNN on a visual object recognition using the ILSVRC2012 ImageNet dataset (Russakovsky et al., 2015). The network's object classification performance achieved a significant accuracy of 55.23% (1,000 classes; chance level, 0.1%).

**Figure 5.**
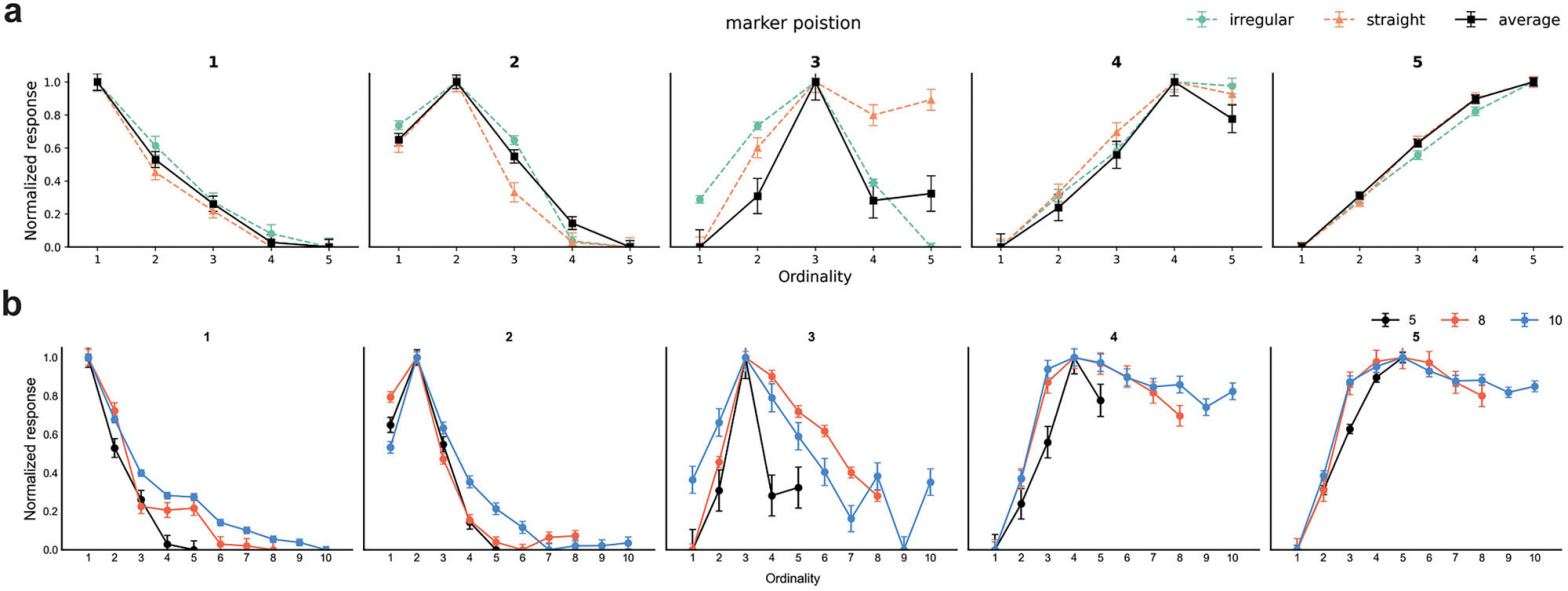
Ordinality-tuned network units. ***a***, Average tuning curves of ordinality-selective network units tuned to each ordinality. Colored curves show the average responses for each stimulus set. Black curves show the average responses over all stimulus set. Error bars indicate SE measure. ***b***, Similar tuning curves were found when evaluating the response of the network units on longer shapes with more ordinalities (maximal ordinality of 8 or 10; colored curves).

We tested if the natural image object classification network could process ordinal information by observing its internal activations to varying ordinalities. A two-way ANOVA with ordinality and stimulus set as factors identified 345 units exhibiting a significant main effect of ordinality (*p* < 0.05), while no significant main effect of stimulus set or interaction effect was observed, revealing selectivity for ordinality. The maximal responses within the selective unit population, indicative of preferred ordinality, were distributed across all presented ordinal values (1–5), with a prominent number of units exhibiting a preference for the first position (preferred ordinality 1, 50.72%; 2, 12.17%; 3, 3.18%; 4, 1.16%; 5, 32.75%). We average and normalized (0–1 range) the responses of ordinality-selective units sharing the same preferred ordinality to generate pooled network tuning curves. The pooled network units demonstrated clear tuning characteristics, with each unit exhibiting a peak response at its preferred ordinality and a decrease in response as the presented ordinality deviated from this optimum (Fig. 5*a*).

We then tested if these ordinality-selective units could perform ordinality comparisons. Given a bias toward the “first” ordinality in the units' distribution, we trained a support vector machine (SVM) to differentiate responses to “first” versus all other ordinalities using a new set of snake images. The SVM achieved significant classification accuracy (mean 0.54, *p* = 0.031, Wilcoxon signed-rank test, one-tailed, *n* = 5 cross-validations).

We then explored the influence of stimulus length on the unit's response by generating longer snake-like stimuli composed of 16 or 20 blocks (compared with the original 10 blocks). These stimuli maintained the visual features of the initial set (irregular/straight shape, varied circle/rectangle placements; see Materials and Methods) but increased the maximal ordinality (8 and 10). We measured the responses of the 345 units to a set of 640 16-block snake images and 800 20-block snake images. Despite the change in stimulus length, averaging the normalized responses based on each unit's maximum activation revealed consistent tuning for ordinalities 1 to 5 (Fig. 5*b*).

To control for the potential confound between ordinality and the distance from the stimulus's starting point, we introduced a control condition in our CNN analyses where compartment lengths varied (Text S3, Fig. S6*a*). This manipulation dissociated ordinal position from starting point distance. Despite these variations, the ordinality-tuned neural units maintained their original tuning patterns (Fig. S6*b*). Moreover, similar tuned responses were observed when the network was tested on the fMRI dataset (Fig. S6*c*), supporting the generalization of ordinality tuning across stimulus types.

Last, we fitted Gaussian functions to the network tuning curves, taking 15 curves in total (ordinalities 1–5 for each of the three stimulus lengths). We measured the tuning curve width as a factor of preferred ordinality. As was found in the neural populations recorded in the cortex, the fitted Gaussians increased in size as a function of ordinality (Kendall's tau one tail; *t*_(15)_ = 0.3714, *p* value: 0.0295).

## Discussion

We identified neuronal populations in the human brain tuned to nonsymbolic ordinal positions. This was supported by similarly tuned units in a biologically inspired convolutional neural network. As in other sensory domains, tuning width increased with preferred ordinal rank, suggesting reduced encoding precision—and potentially lower perceptual accuracy—for higher ranks. Additionally, pRF measurements revealed that cortical territory devoted to higher ordinalities decreased with rank, reinforcing the idea that neural precision is greatest for early positions and declines with increasing ordinal position. Together, these results suggest that the tuning properties support nonsymbolic ordinality perception, akin to tuning observed in other forms of quantity representation (Jacob and Nieder, 2008; Nieder and Dehaene, 2009; Kersey and Cantlon, 2017; Lasne et al., 2019; Nieder, 2020, 2005; Tsouli et al., 2022)

We find the spontaneous emergence of nonsymbolic ordinality-tuned neural units in a network that mimics the visual system and was trained on visual classification. This result supports the existence of inherent ordinality-tuned neurons. The notion that tuning characteristics underlie nonsymbolic ordinality perception is in line with behavioral measures in both animals and humans. For example, Weber's law was found to be a predictor of accuracy in monkeys trained on the ordinality of numerical magnitudes (Brannon and Terrace, 1998; Brannon et al., 2006). Moreover, guppies, trained to identity a feeder within a series of identical ones, were found to make more mistakes when the trained feeder was the fifth and not the third (Petrazzini et al., 2015). In humans, the findings of the canonical distance effect between nonsymbolic stimuli (Lyons and Beilock, 2013; Vogel et al., 2017) and symbolic stimuli (Hamilton and Sanford, 1978; Turconi et al., 2004; Attout et al., 2014; Goffin et al., 2020) are consistent with neuronal tuning underlying perception.

Could the pRF ordinality tuning be driven by numerosity and numerosity tuning? Specifically, may the participants have counted position changes relative to each other? We don't think this is the case. While counting may have been a strategy in the ascending sequence, it is much less likely in the descending sequence. Another possible task strategy could involve implicitly estimating the total number of segments up to the target, effectively turning the task into a numerosity estimation task. However, ordinality tuning was broader, covered smaller cortical areas, and lacked topographically organized, which sets it apart from numerosity tuning (Harvey et al., 2013; Harvey and Dumoulin, 2017; Cai et al., 2021). Finally, artificial neural networks showed units sensitive to numerosity and ordinality but with little overlap, supporting the notion of different networks for numerosity and ordinality.

The ordinality pRF tuning is wider than we typically find in numerosity tuning (for mean tuning curves per ordinality, see Fig. S2). This may suggest that individual neurons are coarsely tuned for ordinality. Alternatively, this may also be explained by individual neurons with more precise tuning but that a diverse neuronal population with different preferred tunings contributes to the population tuning, effectively increasing the population tuning width. Furthermore, we did not find an organized pattern of preferred ordinalities (e.g., topographic maps). This cortical representation, together with the resolution limits of our data acquisition, may further inhibit our ability to precisely measure the width of the tuned response. With the current data we cannot distinguish these two hypotheses.

We found fewer neural populations tuned for higher ordinalities in the cortex, and ordinality tuning was most robust for ordinalities 1–4. The low number of data points tuned to high ordinalities may be explained by a cortical magnification factor for ordinality, akin to primary sensory cortices (Penfield and Boldrey, 1937; Daniel and Whitteridge, 1961; Hubel and Wiesel, 1974) and numerosity (Harvey et al., 2013). Moreover, the absence of spatial topographic organization limited our ability to detect small groups of neurons tuned to higher ordinalities. We do not exclude that neurons are tuned to higher ordinalities but that these represent a minority and hence contribute less to the population tuning. The low number of data points with preference to ordinalities above 4 may reduce the robustness of the measured tuning width, producing outliers in the analysis of their correlation.

The distribution of ordinality-selective tuning curves provides evidence for a primacy effect in ordinality perception. The strong bias toward the first ordinal position highlights an increased importance of the initial parts of an ordered sequence, much like the enhanced recall of the first items in a list (Murdock, 1962).

We did not observe that the ordinality-tuned neuronal populations were organized in topographic maps. In the case of ordinality, similar to other quantity domains, these topographic representations must emerge during development, e.g., due to efficiency in wiring (Cajal, 1999; Chen et al., 2006). On the one hand, not all neurons with tuning properties are organized in topographic maps, but rather in columnar structures (Mountcastle, 1957; Hubel and Wiesel, 1977). On the other hand, we may not have been able to reconstruct topographic maps predominantly due to methodological limitations such as spatial resolution.

Could the ordinality tuning be related to distance from the starting point rather than ordinality per se? Geodesic distance along the snakes is indeed correlated with ordinality. However, Euclidian distance does not perfectly align with ordinality due to the random curvatures of the snake. For example, in some configurations, the 3rd and 5th positions were in the same Euclidian distance from the 1st position. Though we believe Euclidean distance cannot explain ordinality tuning, perhaps distance along the snake could explain ordinality tuning rather than ordinality per se? To control for distance along the snake, we also introduced a control condition in our CNN analyses where the stimuli's compartment lengths varied, resulting in different distances from the starting point to the same ordinal positions (Fig. S6*a*). Despite these variations, the ordinality-selective CNN units exhibited consistent tuning patterns (Fig. S6*b*). These findings suggest that neither Euclidean distance nor distance along the snake underlie the observed tuning.

Similarly, could the ordinality tuning be related to a task component, such as working memory? The participants judged when the marker moved one step forward and no ordinality judgments were required. The memory load was little and identical for different marker positions. We have recently used an identical task in numerosity (Cai et al., 2023). The results were different from the results here. This indicates that this task design, or at least the memory component alone, does not underlie the results. The fact that ordinality-tuned neural units spontaneously developed in the HCNN after object recognition training strongly suggests that these responses are a fundamental property and not merely a consequence of the particular task or its working memory demands.

We report on ordinality-tuned neuronal populations for nonsymbolic ordinality predominantly in the intraparietal sulcus and the premotor cortex. These same cortical regions were also found to be involved in ordinality judgments of nonsymbolic (e.g., shapes at different sizes) and symbolic stimuli of learned ordered sequences (numbers, letters, or months of the year; Fulbright et al., 2003; Fias et al., 2007; Ischebeck et al., 2008). However, shared cortical regions do not necessarily imply the same neuronal populations (Hofstetter et al., 2021). In line with the notion of overlapping but distinct neuronal populations, our results suggest that the neuronal populations selective to nonsymbolic ordinality do not generalize their response to ordinality represented by symbols (letters). Similarly, Zorzi et al. (2011) found different types of symbolic representation (numbers and letters) might also be represented by distinct activity pattern within the same cortical area. Therefore, though similar cortical areas are involved in nonsymbolic and symbolic ordinality, the underlying neuronal populations may be distinct.

The tuned responses to nonsymbolic ordinality we found here show that this type of ordinality representation can occur immediately and do not require training. Specifically, the “snake” shapes stimuli did not involve any sequence learning nor relied on the memory of over-learned sequences (Fig. 1). Therefore, the pRF ordinality-tuned responses may be distinct from previous studies in monkeys that found neuronal selectivity for the temporal order of learned visual lists (Ninokura et al., 2004) or for the ordinal position of an action or movement in a task-related motor sequences (Funahashi et al., 1997; Shima and Tanji, 2000; Berdyyeva and Olson, 2010). However, we do not suggest that the tuned ordinality responses are an abstract cognitive representation of ordinality. It is highly likely that these responses are visually dependent and, to some extent, task dependent (i.e., they rely on the simultaneous presentation of ordered stimuli rather than a temporal sequence).

In summary, the ability to represent the ordinal relation between items seems to be inherent in us. Ordinality provides additional layer of information to help navigate the surrounding environment. The findings of correlations between ordinality processing and arithmetic abilities in adulthood (Lyons and Beilock, 2011; Goffin and Ansari, 2016; Vogel et al., 2017; Vos et al., 2017) and in development (Lyons et al., 2014; Lyons and Ansari, 2015) strengthen the importance of this function. Our findings of ordinality-tuned neuronal responses to nonsymbolic ordinality help to shed light on the underlying neuronal processing of ordinality in the brain.
